# Physical activity and sedentary behavior among adolescents in rural South Africa: levels, patterns and correlates

**DOI:** 10.1186/1471-2458-14-40

**Published:** 2014-01-16

**Authors:** Lisa K Micklesfield, Titilola M Pedro, Kathleen Kahn, John Kinsman, John M Pettifor, Stephen Tollman, Shane A Norris

**Affiliations:** 1 MRC/Wits Developmental Pathways for Health Research Unit, Department of Paediatrics, Faculty of Health Sciences, University of the Witwatersrand, Johannesburg, South Africa; 2 MRC/Wits University Rural Public Health and Health Transitions Research Unit (Agincourt), School of Public Health, Faculty of Health Sciences, University of the Witwatersrand, Johannesburg, South Africa; 3 Umeå Centre for Global Health Research, Epidemiology and Global Health Unit, Department of Public Health and Clinical Medicine, Umeå University, 901 85, Umeå Sweden; 4 INDEPTH Network, Accra, Ghana

**Keywords:** Rural, Sedentary, South Africa, Adolescent, Sport, Physical activity

## Abstract

**Background:**

Physical inactivity is increasing among children and adolescents and may be contributing to the increasing prevalence of overweight and obesity. This study examines physical activity and sedentary behavior patterns, and explores associations with individual, maternal, household, and community factors amongst rural South African adolescents.

**Methods:**

In 2009, 381 subjects, stratified by ages 11-12-years and 14-15-years, were randomly selected from 3511 children and adolescents who had participated in a growth survey two years previously. Weight and height were measured and self-reported Tanner pubertal stage was collected. A questionnaire quantifying frequency and duration of physical activity (PA) domains and sedentary time for the previous 12 months was administered. Moderate-vigorous physical activity (MVPA mins/wk) was calculated for time spent in school and club sport. Socio-demographic and other related data were included from the Agincourt health and socio-demographic system (HDSS). The Agincourt HDSS was established in 1992 and collects prospective data on the community living in the Agincourt sub-district of Mpumalanga Province in rural north-east South Africa.

**Results:**

Puberty, maternal education and socio-economic status (SES) contributed significantly to the mulitiple linear regression model for sedentary behavior (R^2^ = 0.199; adjusted R^2^ = 0.139; p < 0.000), and sex, SES and maternal education contributed to the tobit regression model for school and club sport MVPA (p < 0.000). MVPA, calculated from school and club sport, was higher in boys than girls (p < 0.001), and informal activity was lower (boys: p < 0.05 and girls: p < 0.01) while sedentary time was higher (girls: p < 0.01) in the older than the younger groups. Ninety-two percent (92%) of the sample reported walking for transport.

**Conclusions:**

In this study of rural South African adolescent boys and girls, SES at the maternal, household and community level independently predicted time spent in sedentary behaviors, and school and club MVPA. This study provides local data that can be used to develop health promotion strategies specific to this community, and other similar communities in developing countries.

## Background

Physical inactivity is increasing amongst children and adolescents, with many studies in high-income countries reporting a consequent increase in overweight and obesity [1]. In low- and middle-income countries (LMICs), urbanization and the nutrition transition have been shown to be largely responsible for the increase in overweight and obesity, with recent research from a poor rural South African setting reporting a prevalence of combined overweight and obesity of 15-25% among participants between the ages of 10 and 20 years [2], a finding that is higher than anticipated, but still lower than in urban South African adolescents [3].

International data published in the recent Lancet Physical Activity Series [4] reports that 80% of 13–15 year olds do not meet the current physical activity recommendations of 60 minutes of moderate to vigorous physical activity per day, and also highlights the need for more physical activity surveillance data from Africa. The prevalence of physical inactivity, defined as doing no or very little physical activity at work, at home, for transport or during discretionary time, has been estimated to be 43%–49% in South Africans 15 years of age and older [5], and it has been reported from other African countries that less than 50% of adolescents between 13 and 15 years of age are physically active for at least 60 minutes a day on at least 3 days a week [6]. Objective measures of physical activity in rural-dwelling South African men and women confirmed the significant contribution of ambulatory (walking) activity to overall physical activity in this population [7], and the association between ambulatory activity and adiposity [8] provides more reason for why it is important to understand factors, specific to rural South African adolescents, associated with physical activity.

A socio-ecological approach to physical activity, as described by Bauman et al., [9], which aims to understand how it is influenced by multiple factors operating at different interaction levels, will assist in designing effective interventions that promote physical activity in this population. Therefore the aim of this study is to examine physical activity patterns, and to explore the association between socio-ecological factors (individual, maternal, household and community), and time spent in sedentary behavior, informal, ambulatory and organized physical activity, in a sample of young adolescents living in rural South Africa, a country currently undergoing rapid health transitions.

## Methods

### Subjects

This cross-sectional study was nested within the Agincourt Health and Socio-demographic Surveillance System (AHDSS), which has been described in detail previously [10,11]. In 2009 a sub-sample of 600 participants from three age groups (7–8 years, 11–12 years and 14–15 years) were randomly selected from 3511 children and adolescents who had participated in a 2007 growth survey in the Agincourt sub-district of Mpumalanga Province [2]. The original 2007 growth study randomly selected children and adolescents between the ages of 1 and 20 years (~ 100 boys and 100 girls for each year of age) who had lived in the study area at least 80% of the time since birth, or since 1992 when enrolment into the Agincourt HDSS begun. A random sample of children was drawn from each age-sex-village stratum in proportion to the population size of the village.

For this study 381 subjects, stratified by ages 11–12 years (younger group; YG) and 14–15 years (older group; OG), on whom physical activity data was collected, were included. To ensure that this sub-sample was representative of the larger 2007 study sample [12] we compared various SES parameters between the samples, and found no differences (data not shown). Data for the 7–8 year old age group was not included in this study due to their poor comprehension and understanding of the physical activity questions.

## Ethical approval

Ethical approval was granted by the University of the Witwatersrand Committee for Research on Human Subjects (# M090212), and from the Mpumalanga Provincial Government’s Department of Health, and therefore this study has been performed in accordance with the ethical standards laid down in the 1964 Declaration of Helsinki and its later amendments. Furthermore, permission from the community leaders and school principals was obtained. Parental consent, as well as participant assent was secured after full explanation of the study objectives and testing procedures.

### Participant measurements

Anthropometry: Height (in mm) was measured using a stadiometer (Holtain, UK) and converted to metres (m), and weight was measured to the nearest 0.1 kg using an electronic bathroom scale. All participants were measured wearing light clothing and without shoes. BMI was calculated as weight in kilograms (kg) /height (m)^2^. Age- and sex-specific cut-offs for overweight and obesity using BMI, as recommended by the International Obesity Taskforce [13] were used to determine the prevalence of overweight and obesity These cut-offs have been shown to be applicable to the South African population [2].

Pubertal stage: Pubertal assessment was completed using the Tanner 5-point pubertal self rating scale which has been validated previously for black South Africans [14], and is included to determine the contribution of pubertal development to changes in physical activity levels and patterns. Genital development in boys and breast development in girls were used to define pubertal stages, with stages ranging from stage 1 (pre-pubertal) through to stage 5 (post-pubertal). Tanner stage 1, 2–4 and 5 were defined as pre-pubertal (PreP), mid-pubertal (MP) and post-pubertal (PP), respectively. As there were only 2 boys who were classified as PP, comparison of the physical activity variables was only completed between the boys in the PreP and MP stages.

Physical activity: A questionnaire quantifying total physical activity (PA) for the previous 12 months was administered via interview. The questionnaire was developed to be appropriate for South African children, and has been used on an urban South African cohort at 9 years of age [15]. The questionnaire has also recently been validated in a South African population [16]. Reported frequency and duration of all physical activities (physical education, extra-mural school and club sport, informal physical activity, and walking to and from school) and sedentary activities were recorded. School sport was defined as any extra-mural sport organized by the school, and club sport was defined as private extra-mural activities, such as club soccer. Informal activities included play activities at home or in the neighborhood outside school such as skipping, playing ball games and dancing. Sedentary activities included watching television, playing computer games, listening to music, playing a musical instrument, and reading and drawing. To determine the intensity of the school and club sports, the metabolic equivalent (MET, one MET is defined as the energy expenditure for sitting quietly, which for the average adult is approximately 3.5 ml of oxygen/kg body weight/min) of the various sports reported by the participants was determined according to the classification by Ainsworth et al. [17]. Vigorous sports (≥ 7 METs) included soccer and hockey, while moderate intensity sports (3–6 METS) included netball and athletics. Moderate-vigorous physical activity (MVPA) was calculated by adding all the time spent in moderate and vigorous school and club sport.

A variety of socio-demographic and other related data were included from the growth survey conducted two years previously. These included data about the participants’ mothers (age, nationality, highest education level, marital/union status and whether she resides with the participant or not), their household head, or the individual in the house who provides actual support and maintenance and takes responsibility for one or more individuals in the household (age, sex and highest education level of the household head, household food security and socio-economic status (SES)), and their community (whether the village they lived in was inhabited predominantly by South African or Mozambican people). The Mozambicans in Agincourt are self-settled former refugees who entered South Africa during and after the civil war in the early- to mid-1980’s and elected to stay [10]. These variables have been described in detail previously [12].

### Statistical analysis

Data are presented as mean ± SD for subject characteristics and other normally distributed data, and as median (inter-quartile range) for physical activity parameters and non-normally distributed data. The boys and girls were combined and bivariate linear regression analyses were conducted for both age groups, with individual, maternal, household and community factors as explanatory variables, and the physical activity variables as outcome variables. Multivariate regression analyses were completed to determine the most significant contributors from the significant individual, maternal, household and community factors, to the different physical activity parameters including informal activity, sedentary activity, and walking for transport in the combined age groups. A tobit regression model was completed for school and club MVPA mins/wk to accommodate participants with zero values. Comparisons between sex and age groups were completed using ANOVA when data was normally distributed (age, weight, height and BMI) and Mann–Whitney U-test for non-normally distributed data (all physical activity parameters). Kruskal-Wallis statistics were used to compare physical activity data between 3 or more groups. Chi-square was used to compare proportions. A two-sided P value of <0.05 was considered statistically significant.

## Results

### Subject characteristics

The younger age group (YG: 11–12 years; mean age 12.1 ± 0.6 years) comprised 98 boys and 97 girls (Table 1). The girls were significantly heavier, taller, and had a greater BMI than the boys. In addition, there was a significant difference in pubertal development between the boys and girls in this group. Twelve girls (12.4%) were identified as overweight and 4 girls (4.1%) as obese, and 3 boys (3.1%) as overweight and 1 boy (1%) as obese.

**Table 1:** Subject characteristics of 11–12 and 14–15 year old boys and girls

	**11-12 years**		**14-15 years**	
	**Boys (n = 98)**	**Girls (n = 97)**	**Boys (n = 91)**	**Girls (n = 95)**
Age (yrs)	12.1 ± 0.6	12.1 ± 0.6	15.1 ± 0.7	15.1 ± 0.6
Weight (kg)	35.8 ± 6.8^c^	40.7 ± 10.6^c^	50.7 ± 10.0^A^	55.2 ± 11.2^A^
Height (cm)	145.0 ± 7.1^c^	148.8 ± 7.9^c^	163.1 ± 8.7^B^	159.8 ± 5.5^B^
BMI (kg/m^2^)	16.9 ± 2.0^b^	18.2 ± 3.7^b^	18.9 ± 2.4^C^	21.6 ± 4.1^C^
Pubertal stage (n,%)				
Tanner 1	42 (43%)^b^	20 (21%)^b^	10 (11%)^B^	1 (1%)^B^
Tanner 2	33 (34%)	45 (47%)	11 (12%)	5 (5%)
Tanner 3	19 (19%)	29 (30%)	34 (38%)	40 (43%)
Tanner 4	4 (4%)	3 (3%)	32 (36%)	41 (44%)
Tanner 5	0 (0%)	0 (0%)	2 (2%)	7 (7%)

All values are means ± standard deviation. Comparisons between sex and age groups were completed using ANOVA when data was normally distributed and Mann–Whitney U-test for non-normally distributed data. ^a^P < 0.05, ^b^P < 0.01 and ^c^p < 0.001 for boys vs. girls in the 11-12-year age group; ^A^P < 0.05, ^B^P < 0.01 and ^C^p < 0.001 for boys vs. girls in the 14-15-year age group.

The older age group (OG: 14–15 years; mean age 15.1 ± 0.6 years) comprised 91 boys and 95 girls (Table 1). As with the YG, the girls were heavier and had a greater BMI than the boys, however the boys were significantly taller than the girls. Pubertal development was also greater in the girls compared to the boys, with approximately 50% of the girls nearing the end of puberty (Tanner stages 4 or 5) compared to only 38% of the boys (p < 0.01). Eighteen girls (18.9%) were identified as overweight and 3 girls (3.2%) as obese, and one boy (1.1%) was identified as overweight, and 2 boys (2.2%) as obese.

### Physical activity: sex differences

Physical activity parameters for the two age groups are presented in Table 2. In the younger group, 56% of boys and 65% of girls reported participating in physical education at school, and there was no significant difference in the time (mins/wk) spent in physical education, informal activity or sedentary behavior between the sexes at this age. Significantly more boys than girls (97 vs. 89%; p < 0.05) reported walking to and from school, however there was no difference in the time spent walking between the boys and girls who walked (median, IQR: 200, 100–300 mins/wk). Less than 5% of boys and girls reported cycling to and from school. More than half (56%) of the boys participated in club sport compared to only 25% of the girls. More girls than boys participated in moderate intensity school and club sports while more boys than girls participated in vigorous intensity school and club sports. Time spent in vigorous school sport and total school and club MVPA mins/wk were significantly higher in the boys compared to the girls.

**Table 2:** Physical activity parameters in 11–12 and 14–15 year old boys and girls

	**11-12 years**		**14-15 years**	
	**Boys (n = 98)**	**Girls (n = 97)**	**Boys (n = 91)**	**Girls (n = 95)**
PE (Y,%)	56	65	63	67
PE (mins/wk)	30 (0–60)	30 (0–120)	30 (0–60)	40 (0–60)
Informal (mins/wk)	540 (280–1140)^d^	570 (210–1020)^E^	420 (175–840)^d^	360 (130–570)^E^
Sedentary (mins/wk)	1020 (690–1304)	840 (600–1380)^E^	1035 (740–1440)	1200 (780–1604)^E^
Walking to and from school (mins/wk)*	200 (100–300)	200 (100–300)	200 (100–300)	200 (100–300)
Moderate SS:				
Y,%	39	60	38	51
mins/wk*	62.5 (24–96)	48 (24–135)	36 (24–90)	60 (30–101)
Vigorous SS:				
Y,%	66	16	73	33
mins/wk*	72 (45–144)^b^	24 (12–72)^b,E^	81 (36–192)^B^	36 (15–96)^B,E^
Moderate CS:				
Y,%	6	22	10	21
mins/wk*	57 (12–108)	72 (48–144)	60 (30–120)	66 (21–132)
Vigorous CS:				
Y,%	56	7	69	20
mins/wk*	150 (96–360)^e^	108 (45–216)	288 (90–540)^B,e^	36 (20–75)^B^
MVPA	251 (84–420)^c.d^	60 (27–210)^c^	360 (135–648)^C,d^	81 (33–192)^C^
(mins/wk)*	n = 86	n = 63	n = 83	n = 67

Data presented as median (interquartile range). Comparisons between sex and age groups were completed using ANOVA when data was normally distributed and Mann–Whitney U-test for non-normally distributed data. Y,%: percentage of participants who answered yes. *Data only for subjects who reported participating in these activities. SS: school sport; CS: club sport; MVPA: school and club moderate-vigorous physical activity. ^a^P < 0.05, ^b^P < 0.01 and ^c^p < 0.001 for boys vs. girls in the 11-12-year age group; ^A^P < 0.05, ^B^P < 0.01 and ^C^p < 0.001 for boys vs. girls in the 14-15-year age group; ^d^P < 0.05, ^e^P < 0.01 and ^f^p < 0.001 for differences between the age groups for boys; ^D^P < 0.05, ^E^P < 0.01 and ^F^p < 0.001 for differences between the age groups for girls.

There was no difference between the number of 14–15 year old boys compared to girls participating in physical education (63 vs. 67%) and the time (mins/wk) spent in physical education at school. There were no differences between the sexes in time spent participating in informal activity or sedentary behavior, or time spent walking to and from school in this age group. More boys than girls reported cycling to school (8 vs. 2%), while 90 and 94% of the boys and girls, respectively reported walking to and from school. Significantly more boys than girls reported participating in club sports (70 vs. 33%; p < 0.0001), and the sport most commonly reported in the boys was soccer and in the girls was netball. More girls than boys reported participating in moderate intensity school and club sport, while more boys than girls reported participating in vigorous intensity school and club sport. In the subjects who reported participating in school and club sport, total MVPA mins/wk was significantly higher in the boys compared to the girls.

### Physical activity: age differences

Compared to the younger boys, the older boys reported spending significantly less time in informal activity (p < 0.05), but more of them reported participating in vigorous club sport and for a significantly greater amount of time (p < 0.01). In those boys who participated in school and club sport, MVPA mins/wk was significantly higher in the older boys compared to the younger boys (p < 0.001).

Compared to the younger girls, the older girls reported spending less time in informal activity (p < 0.01), more sedentary time (p < 0.01) and more time in vigorous intensity school sport (p < 0.01).

### Physical activity: pubertal differences

In the boys and girls, increasing pubertal development was associated with an increase in sedentary time (p < 0.001), however none of the other physical activity variables were different between the pubertal groups.

### Correlates of physical activity

Maternal, household and community data collected two years previously has been described for the larger growth survey [12], and is presented for this sample in Table 3. Significant predictors of the physical activity domains were identified at the individual, maternal, household and community levels.

**Table 3:** Maternal, household and community characteristics of boys (n = 189) and girls (n = 192), Agincourt 2007

	**Boys**		**Girls**	
	**n**	**%**	**n**	**%**
**Maternal characteristics** (2 years previously)				
Age (years)				
24–34	58	36.3	47	29.9
35–49	69	46.3	92	58.6
50+	22	14.8	18	11.5
Education				
No formal education	38	29.5	39	30.2
Primary education	36	27.9	33	25.6
Secondary education	46	35.7	51	39.5
Higher than secondary	9	7.0	6	4.7
Nationality				
South African	100	67.1	102	65
Mozambican	49	32.9	55	35
Marital/union status				
Currently in union	86	53.8	90	53.3
Not in union	74	46.3	79	46.7
Co-residence with child				
Alive, co-residing	134	83.8	137	81.1
Alive, not co-residing	14	8.8	21	12.4
Dead	12	7.5	11	6.5
**Household characteristics** (2 years previously)				
Age of household head (years)				
15–34	7	4.4	4	2.4
35–49	65	40.6	68	40.2
50+	88	55	97	57.4
Sex of household head				
Male	106	66.3	118	69.8
Female	54	33.7	51	30.2
Household head education				
No formal education	69	48.6	73	48.7
Primary education	47	33.1	39	26
Secondary education	25	17.6	33	22
Higher than secondary	1	0.7	5	3.3
Household head relationship to child				
Parent	99	62.7	107	63.3
Grandparent	49	31.0	52	30.8
Other	10	6.3	10	5.9
Enough food				
Yes, food secure	127	81.4	138	82.6
No, food insecure	29	18.6	29	17.4
SES (wealth index tertile)				
Lowest	54	34.0	61	36.3
Medium	60	37.7	49	29.2
Highest	45	28.3	58	34.5
**Community characteristics** (2 years previously)				
Area of residence				
Predominantly South Africa	151	94.4	155	91.7
Predominantly Mozambican	9	5.6	14	8.3

### Individual characteristics

Neither sex nor pubertal status significantly predicted time spent in informal activity or walking for transport in either age group. In both age groups, being a girl was associated with approximately 3–5 hours less school and club MVPA mins/wk (both p < 0.001). Compared to PreP adolescents, MP adolescents reported significantly more sedentary time per week in the younger group (p < 0.01) and the older group (p < 0.05). In the older group only, PP adolescents reported approximately 13 hours more sedentary time per week compared to the PreP group (p < 0.01). In the younger group, MP reported participating in significantly less school and club MVPA mins/wk compared to PreP adolescents (p < 0.01).

### Maternal characteristics

Maternal age was not associated with any of the physical activity domains in either age group, however in the younger group the children of Mozambican mothers reported less sedentary time than the children of South African mothers (p < 0.05). Compared to children whose mothers had no education, children whose mothers had a secondary school education or higher, reported significantly more sedentary time (both age groups p < 0.01), and significantly less walking for transport (YG: p < 0.05; OG: p < 0.01). In the older group, higher maternal education was associated with nearly three hours more school and club MVPA per week (p < 0.05).

### Household characteristics

In both age groups, children living in households where the household head had completed secondary school reported spending approximately five hours more in sedentary activity than the children living in households where the household head had no education (both p < 0.001). Homes with a female household head predicted less sedentary activity in the younger group, compared to homes in which the household head was male (p < 0.05). In the older group, the children who resided in homes in which the household head had completed primary school reported more informal activity (p < 0.05) and more school and club MVPA per week (p > 0.001) compared to children residing in homes in which the household head had no education. In the older group, children in the highest SES tertile spent 2 hours/wk more in school and club MVPA compared to children in the lowest SES tertile (p = 0.05).

### Community characteristics

In the younger group, children who were from villages inhabited predominantly by people of Mozambican origin reported nearly four hours less sedentary activity per week compared to children from villages inhabited by predominantly local South Africans (p < 0.01).

### Multivariate regression models for physical activity parameters

All individual, maternal, household and community variables were entered into a multiple regression to explain the various physical activity domains. Only multiple regression models for sedentary time and school and club MVPA reached significance; these are presented in Tables 4 and 5, respectively. Factors that contributed significantly to the model for sedentary time (Table 4: R^2^ = 0.199, adjusted R^2^ = 0.139, p < 0.000) included pubertal stage, which showed that when compared to the PreP group, the MP and PP groups reported 304 and 732 more sedentary minutes per week, respectively. Higher maternal education, a proxy for SES, was associated with more than four hours of sedentary time, however children in the middle SES tertile participated in less sedentary time than children in the lowest tertile.

**Table 4:** Multivariate regression model for sedentary behavior (mins/wk)

	**Coefficient**	**SE**	**95% CI**	**P**
**Individual characteristics**				
Age	29.3	26.4	-23, 81	0.267
BMI	6.3	12	-17, 30	0.600
Sex				
Boy (ref)	0			
Girl	-117	76.5	-267, 34	0.129
Pubertal status				
Pre-pubertal (ref)	0			
Mid-pubertal	304	102	103, 505	**0.003**
Post-pubertal	732	257	226, 1238	**0.005**
**Maternal characteristics**				
Age	4.6	5.2	-6, 15	0.371
Nationality				
South African (ref)	0			
Mozambican	63.8	101.5	-136, 264	0.530
Education				
Pre-school (ref)	0			
Primary school	-30	113.4	-254, 193	0.790
Secondary school	265.6	134.7	0.17, 531	**0.050**
**Household characteristics**				
Sex				
Male	0			
Female	-155.5	80.5	-314, 3	0.055
Education				
Pre-school (ref)	0			
Primary school	125.7	95	-61, 313	0.187
Secondary school	185.2	112.1	-36, 406	0.100
Food security				
Yes (ref)	0			
No	34	95.2	-154, 222	0.722
SES (wealth index tertile)				
Lowest (ref)	0			
Medium	-195	93.4	-379, -11	**0.038**
Highest	-96	98.6	-290, 99	0.332
**Community characteristics**				
Area of residence				
Predominantly South African (ref)	0			
Predominantly Mozambican	98.6	150	-198, 395	0.512

*SE*, standard error; R^2^ = 0.1992; Adjusted R^2^ = 0.1391.

**Table 5:** Tobit regression model for school and club MVPA (mins/wk)

	**Coefficient**	**SE**	**95% CI**	**P**
**Individual characteristics**				
Age category				
11–12 years (ref)	0			
14–15 years	-166	108.2	-379, 47	0.126
BMI	-2.2	6.5	-15, 11	0.732
Sex				
Boy (ref)	0			
Girl	-265	39.5	-343, -187	**0.000**
Pubertal status				
Pre-pubertal (ref)	0			
Mid-pubertal	-73	52	-176, 29	0.161
Post-pubertal	-123	132.6	-385, 138	0.353
**Maternal characteristics**				
Age	-4.1	2.7	-9, 1	0.139
Nationality				
South African (ref)	0			
Mozambican	12.5	52.8	-92, 116	0.813
Education				
Pre-school (ref)	0			
Primary school	-105.5	62.6	-229, 18	0.093
Secondary school	-171.6	79.4	-328, -15	**0.032**
Age category x maternal education	121.7	46.4	30, 213	**0.009**
**Household characteristics**				
Sex				
Male (ref)	0			
Female	25	42	-58, 108	0.552
Education				
Pre-school (ref)	0			
Primary school	66.3	50	-32,165	0.186
Secondary school	5.7	59	-111, 122	0.923
Food security				
Yes (ref)	0			
No	55.7	49.9	-43, 154	0.265
SES (wealth index tertile)				
Lowest (ref)	0			
Medium	63.2	48.9	-33, 160	0.198
Highest	128	51.6	27, 230	**0.014**
**Community characteristics**				
Area of residence				
Predominantly South African (ref)	0			
Predominantly Mozambican	41.4	80.2	-117, 200	0.606

*SE*, standard error.

Significant contributors to the tobit regression model for school and club MVPA (Table 5; log likelihood = -133,3963; p < 0.000) included sex, maternal education and SES tertile, as well as the interaction between age category and maternal education. These data confirmed that boys participated in school and club MVPA mins/wk more than girls, that there was an increase in school and club MVPA mins/wk with SES, and that higher maternal education in the older age group was associated with greater predicted school and club MVPA mins/wk.

## Discussion

In this study of boys and girls living in a poor rural community in South Africa, SES independently predicted sedentary time as well as all physical activity parameters, including informal activity, walking for transport, and MVPA. Lower SES at the maternal, household and community level was significantly associated with less sedentary time, more walking for transport and lower moderate-vigorous physical activity in school and clubs.

The relationship between lifestyle factors, including physical activity, and SES is complex and may differ between LMICs and high income countries (HICs) [18,19]. Our findings indicate that in rural South African adolescents greater SES is associated with more time spent in sedentary behavior’s such as watching television and reading, less time walking as a means of transport, and more time participating in MVPA involved in school and club sports. Although McVeigh et al., [15] have also shown that lower SES is associated with less leisure-time activity in a younger South African cohort, they found that lower SES status was associated with more television time. The difference in these findings may be due to rural children of lower SES in our study needing to assist more with household chores while the children of lower SES living in urban Johannesburg may not have the same responsibilities within the home. Data from the US and Europe have also shown higher SES to be associated with higher levels of moderate to vigorous activity [20,21], however in contrast to our findings, they have shown that increased SES is associated with more self-reported walking [21] and less time spent in sedentary behavior’s such as television-viewing [20]. These findings highlight not only some of the differences in the association between SES and sedentary behavior between LMICs and HICs, but also between urban and rural populations. What appears to be consistent however is that lower SES is associated with reduced moderate to vigorous physical activity; key reasons for this need to be better elucidated to ensure the design of more effective interventions. To our knowledge, this is the first study in South African adolescents to examine the influence of SES on the various physical activity domains; most studies to-date have only measured leisure time activity, and have not taken into account the important contributions of walking for transport and informal activity to overall daily physical activity. Knowledge of time spent in physical activity, as well as physical activity patterns and domains, are important within a public health context in order to identify more precisely the focus of interventions likely to be most effective.

For both boys and girls, the largest proportion of time during the day was spent in sedentary behavior, which for the whole sample accounted for an average of 2–3 hours per day. More than two thirds (68%) of sedentary time was spent in screen time with the remaining time spent doing homework (26%), and listening to the radio (6%). This is in comparison to ~ 25 minutes per day spent in school and club MVPA and 1.5 hours spent in informal physical activity. Although more than 90% of the sample reported walking to and from school, this was for an average of 40 minutes per day. Although only 26% of the sample met the international health guidelines of 60 minutes of MVPA per day (calculated only for school and club sport in this study), the inclusion of the time spent in informal activity (~ 180 mins/day) and walking for transport (~ 65 mins/day) suggest that adolescents in poor rural South African communities are meeting physical activity recommendations. Although comparative data from European adolescents of a similar age report higher values than these, their study also confirms the importance of considering all domains of physical activity [21]. A concern, however, is that a recent systematic review suggests that time spent in sedentary activities, independent of physical activity time, is associated with adverse health outcomes including an increase risk of cardio-metabolic disease in children and adolescents [22]; hence interventions should not focus only on increasing physical activity, but also decreasing sedentary time.

In this study, the increase in female sedentary time with age, as well as pubertal development, mirrors the increase in obesity with age and pubertal development shown previously in this population [12]. As expected due to the relatively low SES of the community in this study, and as has been shown previously in younger rural children [23], the prevalence of overweight (16% for girls and 2% for boys) and obesity (4% for girls and 2% for boys) was lower than the national prevalence data for children of a similar age from the Youth Risk Behaviour Survey [3]. Our finding that greater SES is associated with more sedentary time may further compound the overweight/obesity problems previously attributed to the nutrition transition occurring in LMICs. Further, our data shows that overweight and obesity are no longer limited to urbanized populations as was the case over a decade ago when the prevalence of overweight and obesity was still low in rural South Africa [24], and that rural populations are now also at risk. The findings of this study indicate that increasing sedentary behavior contributes to the development of obesity in adolescent girls, and research on interventions designed to decrease sedentary time in this population are warranted. The increase in sedentary time, particularly in adolescent girls, has been well described in the literature [25,26]. Although the prevalence of overweight and obesity was low in boys, the increase in sedentary time with pubertal development may also have implications for future chronic disease risk in this population. In contrast to other studies [21] we have shown that older boys participated in more school and club MVPA than younger boys, and similarly the older girls spent more time in vigorous physical activity at school than their younger counterparts. These findings may, however, be due to the limitations of self-report and the possibility of over-reporting, known to be affected by social desirability and recall bias.

In this study we measured possible predictors of physical activity at the individual level, and also maternal, household and community factors. The relationship between sedentary time and SES was significant at all levels including maternal (maternal education and maternal nationality), household (household head education) and community (area of residence inhabited predominantly by people of Mozambican or South African people). Gordon-Larsen et al., 2000 [20] have suggested that the determinants associated with physical inactivity are different to those for physical activity, and may be more socio-demographic in nature. The influence of the wider environment on health behavior will also assist in identifying possible areas of intervention and therefore the socio-ecological factors associated with physical activity specific to the population in question must be identified.

Our findings indicate that boys are more physically active than girls in early and mid-adolescence, a finding consistent with data from both LMICs and HICs [21,27]. On average, boys spent 196 minutes a day in physical activity compared to 154 minutes in the girls. In addition we measured the various domains of physical activity and showed that although boys spent more time than girls in vigorous sport, girls spent more time than boys participating in school and club sports of moderate intensity. Although the time and participation in these more formal activities was not different between the age groups for boys and girls, informal activity was lower in the older boys and girls compared to their younger counterparts.

In this study approximately 65% of the participants reported participating in physical education. This is significantly higher than data from an urban sample of 9-year-old children living in Johannesburg, 43% of whom reported participating in physical education at least once a week [28]. In 2010 the Healthy Active Kids South Africa (HAKSA; http://www.globalpa.org.uk/downloads/healthy-active-kids-report-2010.pdf) report card was produced to highlight the health status of the South African youth with regard to major lifestyle risk factors such as smoking, inactivity, obesity and unhealthy eating, and to identify areas for intervention. The HAKSA report card highlights the need for teachers to be more prepared to deliver physical education classes and for more equipment in economically disadvantaged areas, however it is hoped that with recent changes in the curriculum whereby physical education is now a compulsory part of the school week the proportion of children and adolescents participating in regular physical education should increase.

A number of limitations of the study need to be considered. Due to the cross-sectional nature of the data the physical activity differences found between the different age and pubertal groups cannot infer change in physical activity over time, longitudinal cohort data is required to make these inferences. Secondly, we did not collect data on household moderate to vigorous activity which we now see would be of much interest, particularly within a rural context. This study does however provide data on more distal factors (at the level of the mother, household and community) to better understand other variables that are associated with sedentary behavior and physical activity patterns in this sample. This provides more areas for possible intervention strategies.

## Conclusions

Lower SES at the individual, maternal, household and community level was significantly associated with less sedentary time, more walking for transport and lower school and club MVPA. Increased resources, whether through corporate and social investment, NGO initiatives or innovative local efforts, may assist with decreasing sedentary time and increasing the opportunities for moderate and vigorous physical activities.

## Competing interests

The authors declare that they have no competing interests.

## Authors’ contributions

LM performed the statistical analysis and drafted the manuscript; TP collected the data for the study; KK and ST are responsible for the Agincourt Health and Socio-demographic Surveillance System including community engagement; JK, JP and KK provided input on the manuscript; SN conceived of the study and assisted with editing the manuscript. All authors read and approved the final manuscript.

## Pre-publication history

The pre-publication history for this paper can be accessed here:

http://www.biomedcentral.com/1471-2458/14/40/prepub

## References

[B1] Kimm SYS Glynn NW Obarzanek E Kriska AM Daniels SR Barton BA Liu K Relation between the changes in physical activity and body-mass index during adolescence: a multicentre longitudinal study Lancet 2005 366 301 307 10.1016/S0140-6736(05)66837-7 16039332

[B2] Kimani-Murage EW Kahn K Pettifor JM Tollman SM Dunger DB Gómez-Olivé XF Norris SA The prevalence of stunting, overweight and obesity, and metabolic disease risk in rural South African children BMC Public Health 2010 10 158 170 10.1186/1471-2458-10-158 20338024 PMC2853509

[B3] Reddy SP Resnicow K James S Funani IN Kambaran NS Omardien RG Masuka P Sewpaul R Vaughan RD Mbewu A Rapid increases in overweight and obesity among South African adolescents: comparison of data from the South African National Youth Risk Behaviour Survey in 2002 and 2008 Am J Public Health 2012 102 262 268 10.2105/AJPH.2011.300222 21940919 PMC3483977

[B4] Hallal PC Andersen LB Bull FC Guthold R Haskell W Ekelund U Group FTLPASW Global physical activity levels: surveillance progress, pitfalls, and prospects Lancet 2012 380 247 257 10.1016/S0140-6736(12)60646-1 22818937

[B5] Joubert J Norman R Lambert EV Groenewald P Schneider M Bull F Bradshaw D South African Comparative Risk Assessment Collaborating Group Estimating the burden of disease attributable to physical inactivity in South Africa in 2000 S Afr Med J 2007 97 725 731 17952230

[B6] Peltzer K Health behavior and protective factors among school children in four african countries Int J Behav Med 2009 16 172 180 10.1007/s12529-008-9015-3 19424814

[B7] Cook II Alberts MM Brits JSJ Choma SRS Mkhonto SSS Descriptive epidemiology of ambulatory activity in rural, black South Africans Med Sci Sports Exerc 2010 42 1261 1268 20019642 10.1249/MSS.0b013e3181ca787c

[B8] Cook I Alberts M Lambert EV Relationship between adiposity and pedometer-assessed ambulatory activity in adult, rural African women Int J Obes 2008 32 1327 1330 10.1038/ijo.2008.26 18332881

[B9] Bauman AE Reiss RS Sallis JF Wells JC Loos RJF Martin BW Group FTLPASW Correlates of physical activity: why are some people physically active and others not? Lancet 2012 380 258 271 10.1016/S0140-6736(12)60735-1 22818938

[B10] Kahn K Tollman SM Collinson MA Clark SJ Twine R Clark BD Shabangu M Gómez-Olivé FX Mokoena O Garenne ML Research into health, population and social transitions in rural South Africa: data and methods of the Agincourt health and demographic surveillance system Scand J Public Health 2007 35 8 20 10.1080/14034950701505031 PMC2826136 17676498

[B11] Kahn K Collinson MA Gómez-Olivé FX Mokoena O Twine R Mee P Afolabi SA Clark BD Kabudula CW Khosa A Khoza S Shabangu MG Silaule B Tibane JB Wagner RG Garenne ML Clark SJ Tollman SM Profile: agincourt health and socio-demographic surveillance system Int J Epidemiol 2012 41 988 1001 10.1093/ije/dys115 22933647 PMC3429877

[B12] Kimani-Murage EW Kahn K Pettifor JM Tollman SM Klipstein-Grobusch K Norris SA Predictors of adolescent weight status and central obesity in rural South Africa Public Health Nutr 2011 14 1114 1122 10.1017/S1368980011000139 21356151 PMC3370923

[B13] Cole T Bellizzi M Flegal K Dietz W Establishing a standard definition for child overweight and obesity worldwide: international survey BMJ 2000 320 1240 1243 10.1136/bmj.320.7244.1240 10797032 PMC27365

[B14] Norris S Richter L Usefulness and reliability of tanner pubertal self-rating to urban black adolescents in South Africa J Res Adolesc 2005 15 609 624 10.1111/j.1532-7795.2005.00113.x

[B15] McVeigh JA Norris SA Cameron N Pettifor JM Associations between physical activity and bone mass in black and white South African children at age 9 yr J Appl Physiol 2004 97 1006 1012 10.1152/japplphysiol.00068.2004 15132999

[B16] McVeigh JA Norris SA Criterion validity and test-retest reliability of a physical activity questionnaire in South African primary school-aged children J Sports Med 2012 24 43 48

[B17] Ainsworth B Haskell W Whitt M Irwin M Swartz A Strath S O'Brien W Bassett D Schmitz K Emplaincourt P Jacobs D Leon A Compendium of physical activities: an update of activity codes and MET intensities Med Sci Sports Exerc 2000 32 S498 S516 10.1097/00005768-200009001-00009 10993420

[B18] Kim S Contrasting socioeconomic profiles related to healthier lifestyles in china and the United States Am J Epidemiol 2004 159 184 191 10.1093/aje/kwh006 14718221

[B19] Stalsberg R Pedersen AV Effects of socioeconomic status on the physical activity in adolescents: a systematic review of the evidence Scand J Med Sci Sports 2010 20 368 383 10.1111/j.1600-0838.2009.01047.x 20136763

[B20] Gordon-Larsen P McMurray RG Popkin BM Determinants of adolescent physical activity and inactivity patterns Pediatrics 2000 105 e83 10.1542/peds.105.6.e83 10835096

[B21] De Cocker K Ottevaere C Sjöström M Moreno LA Wärnberg J Valtueña J Manios Y Dietrich S Mauro B Artero EG Molnár D Hagströmer M Ruiz JR Sarri K Kafatos A Gottrand F De Henauw S Maes L De Bourdeaudhuij I Self-reported physical activity in European adolescents: results from the HELENA (Healthy Lifestyle in Europe by Nutrition in Adolescence) study Public Health Nutr 2010 14 246 254 20236565 10.1017/S1368980010000558

[B22] Tremblay MS LeBlanc AG Kho ME Saunders TJ Larouche R Colley RC Goldfield G Gorber SC Systematic review of sedentary behaviour and health indicators in school-aged children and youth Int J Behav Nutr Phys Act 2011 8 98 116 10.1186/1479-5868-8-98 21936895 PMC3186735

[B23] Labadarios D Steyn NP Maunder E MacIntryre U Gericke G Swart R Huskisson J Dannhauser A Vorster HH Nesmvuni AE Nel JH The National Food Consumption Survey (NFCS): South Africa, 1999 Public Health Nutr 2005 8 533 543 16153334 10.1079/phn2005816

[B24] Monyeki K van Lenthe F Steyn N Obesity: does it occur in African children in a rural community in South Africa? Int J Epidemiol 1999 28 287 292 10.1093/ije/28.2.287 10342693

[B25] Caspersen CJ Pereira MA Curran KM Changes in physical activity patterns in the United States, by sex and cross-sectional age Med Sci Sports Exerc 2000 32 1601 1609 10994912 10.1097/00005768-200009000-00013

[B26] Telama R Yang X Decline of physical activity from youth to young adulthood in Finland Med Sci Sports Exerc 2000 32 1617 1622 10994914 10.1097/00005768-200009000-00015

[B27] Reddy SP James S Sewpaul R Koopman F Funani NI Sifunda S Josie J Masuka P Kambaran NS Omardien RG Umthente Uhlaba Usamila – The South African Youth Risk Behaviour Survey 2008

[B28] McVeigh J Norris S Wet T The relationship between socio-economic status and physical activity patterns in South African children Acta Paediatr 2007 93 982 988 15303817 10.1111/j.1651-2227.2004.tb02699.x

